# Supplementary material for: Hybrid Models and Biological Model Reduction with PyDSTool
Source: PLoS Comput Biol. 2012 Aug 9;8(8):e1002628. doi: 10.1371/journal.pcbi.1002628 (PMC3415397; doi:10.1371/journal.pcbi.1002628)
Supplement: Text S4 — Complete source code for the PyDSTool package (version 0.88.120504). Includes API documentation and help files linking to web pages. This file is identical to the current public release on Sourceforge.net. (ZIP) [file pcbi.1002628.s004.zip › PyDSTool/html/toc-PyDSTool.Toolbox.dssrt-module.html]

xml version="1.0" encoding="ascii"?


dssrt


# Module dssrt

---

## Classes

EpochSeqScorer  
 Scorer  
 VarAlphabet  
 become\_most\_dominant  
 domscales  
 dssrt\_assistant  
 epoch  
 is\_active  
 is\_fast  
 is\_inactive  
 is\_modulatory  
 is\_most\_dominant  
 is\_order1  
 is\_slow  
 join\_actives  
 join\_fast  
 join\_slow  
 leave\_actives  
 leave\_fast  
 leave\_slow  
 regime  

## Functions

\_comp

as\_absolute\_ratios

as\_relative\_ratios

check\_opts

comp\_seqs  
 define\_psi\_events  
 define\_tau\_events  
 editdist\_edits  
 find\_ep\_ix  
 find\_epoch\_period  
 find\_regime\_transition  
 get\_infs  
 get\_symbol\_sequence  
 get\_taus  
 indent  
 jaro  
 normalized\_psis  

only\_dynamic

pad

partition\_range

plot\_psis  
 show\_epochs  

spectral\_gaps\_absolute

spectral\_gaps\_relative

split

split\_pts  

strip\_speed

swdist  
 tabulate\_epoch\_seqs  
 transition\_psi  
 transition\_tau  

## Variables

APPROX  
 CLOSE  
 FAR  
 GAP  

JARO\_MARKER\_CHAR

MATCH  
 MISMATCH  
 VCLOSE  
 VFAR  

\_classes

\_functions

\_predicates

---

[hide private]
